# A subcellular study on reactive oxygen species generation by PFAS in HepG2 cells

**DOI:** 10.1038/s41598-025-07503-7

**Published:** 2025-07-01

**Authors:** V. H. Amstutz, A. Mircheva, A. Cengo, L. J. Dubois, D. T. H. M. Sijm, M. F. Vrolijk

**Affiliations:** 1 Department of Pharmacology and Toxicology, Faculty of Medicine, Health & Life Science, Maastricht University, PO Box 616, Maastricht, 6200 MD, 6229 ER The Netherlands; 2 Department of Precision Medicine, GROW Research Institute for Oncology and Reproduction, Maastricht University, Maastricht, 6229 ER The Netherlands; 3 Office for Risk Assessment and Research, Netherlands Food and Consumer Product Safety Authority (NVWA), Utrecht, 3540 AA The Netherlands

**Keywords:** Cell biology, Mechanism of action

## Abstract

Per- and polyfluoroalkyl substances (PFAS) hepatotoxicity is well documented, especially for legacy compounds such as PFHxS, PFOA, PFOS, and PFNA. However, the mechanism(s) involved are yet to be fully understood. The present study aims to investigate the origin of PFAS-induced formation of reactive oxygen species (ROS) and their relevance for the decrease of cell viability of HepG2 cells after exposure to PFASs. Moreover, a structure–activity relationship was assessed using PFASs with different headgroups (carboxylic, sulfonic, and alcoholic) and variable carbon-chain lengths (4–10 C). The link between ROS generation and cell viability was assessed using two antioxidants: quercetin, a generic antioxidant, and mito-tempo, a mitochondria-targeted antioxidant. Both antioxidants were demonstrated to be effective in reducing PFAS-induced ROS generation. The mechanism behind PFAS-induced ROS might be headgroup-dependent, as quercetin increased cell viability after both perfluoroalkyl carboxylic acids (PFCA) and perfluorosulfonic acids (PFSA) exposure, while mito-tempo only improved cell viability after PFCAs exposure. The two major sources of ROS generation in HepG2 cells are the peroxisomes and mitochondria. However, exposure to PFASs did not impact peroxisomal or mitochondrial activity after 24 h. Uncommon sources of ROS generation, such as lysosomal leakage or lipid peroxidation, have been demonstrated to result from previously generated ROS and not from PFASs exposure. Indeed, lysosomal leakage caused by PFASs exposure is negated by either quercetin or mito-tempo treatment, while lipid peroxidation only occurs after 24 h of exposure, long after the initial ROS generation by PFASs. This indicates that both events are a result of previously generated ROS. However, exposure to both PFOA and PFOS was demonstrated to reduce catalase activity in HepG2. In conclusion, the present study demonstrates that ROS generation after PFASs exposure might be due to inhibition of HepG2 endogenous antioxidants. Moreover, a headgroup-dependent mechanism of action has been observed, indicating that PFCAs and PFSAs exposure might lead to hepatotoxicity through different pathways.

## Introduction

Per- and polyfluoroalkyl substances (PFASs) have been a concern for human and environmental safety over the last two decades as they have been linked with hepatotoxicity, immunotoxicity, and reprotoxicity^1–3^. However, despite the potential risk to human health, PFASs have become a key component for product manufacturing due to their ability to produce waterproof and stain-resistant surfaces and their high thermal and chemical stability^4^. As a result, PFASs have been employed to produce waterproof fabric, electrical insulators, food contact materials (FCMs), firefighting foam, and personal care products and have become ubiquitous in the human environment^5–7^. Besides the environment, PFASs have also been detected in various human tissues, notably perfluorooctanoic acid (PFOA), perfluorooctansulfonic acid (PFOS), perfluorohexanoic acid (PFHxS), and perfluorononanoic acid (PFNA)^8–11^. The major source of human exposure in the Dutch population appears to be fish and seafood products, followed by warm drinks and fast food^12^.

In response to human exposure to PFASs, several regulatory bodies have tried to regulate their usage. Through the Stockholm Convention on Persistent Organic Pollutants (POPs), the manufacturing and use of PFOA, PFOS, and PFHxS have been restricted^13^. Furthermore, a proposal has been made to phase out the use of the group of over 10,000 PFASs under the REACH regulation^14^ within the European Union. Some industries have responded to those restrictions by replacing the restricted compounds with alternative PFASs that are supposed to be less toxic. Despite these regulatory restrictions, humans are still exposed to restricted, non-restricted, and emerging PFASs, with little information on the safety aspects of most PFASs^15,16^.

Over the years, the toxicity of legacy PFASs, including PFOS and PFOA, has been thoroughly documented and showed a wide range of adverse effects to humans. Notably, hepatotoxicity has been reported in many studies, which, in combination with the exposure levels shown to humans, is relevant to human health. Plasma concentrations of PFHxS, PFOA, PFOS, and PFNA have been associated with the risk of metabolic dysfunction-associated fatty liver disease in male children and of liver steatosis in the exposed population^17–19^. Despite the vast number of publications on the hepatotoxicity of PFASs, the mechanism of action is not yet completely understood. The generation of reactive oxygen species (ROS) following exposure to PFASs has been proposed as a key part of their mechanism of toxicity, notably due to potential disruption in mitochondrial activity^20,21^.

The present study aimed to provide further insights into the mechanism of action by assessing the effect of PFASs on a sub-cellular level in hepatocytes. Specifically, their effects on ROS generation, mitochondrial and peroxisome function, lipid peroxidation, and lysosome membrane integrity were determined. It aimed to assess a structure–activity relationship by selecting PFASs with different headgroups and chain lengths. The selected PFASs were perfluoroalkyl carboxylic acids (PFCA), perfluorosulfonic acids (PFSA), or fluorotelomer alcohols (FTOH) with carbon chain lengths ranging from 4 to 10 carbons.

## Materials and methods

### Materials

The HepG2 cell line was obtained from American Type Culture Collection (ATCC HB8065, Manassas, USA). Dimethyl sulfoxide (DMSO), Dulbecco’s Phosphate Buffered Saline (DPBS), 2,7-dichlorofluorescein-diacetate (DCFH-DA), Triton™ X-100, and hydrogen peroxide (H_2_O_2_) were purchased from Sigma–Aldrich (St Louis, USA). Dulbecco’s minimal essential medium (DMEM), heat-inactivated fetal bovine serum (FBS), penicillin–streptomycin (pen-strep), trypsin–EDTA solution, RIPA lysis buffer, and Pierce BCA protein assay kit were all obtained from Thermo Fisher Scientific (Waltham, USA). The tested PFASs and their respective CAS number are presented in Table 1. Perfluorobutanoic acid (PFBA), PFBS, 2,2,3,4,4,4-Hexafluoro-1-butanol (3:1 FTOH), PFHxA, 3,3,4,4,5,5,6,6,6-Nonafluoro-1-hexanol (4:2 FTOH), Perfluoroheptanoic acid (PFHpA), PFOA, PFOS, 3,3,4,4,5,5,6,6,7,7,8,8,8-Tridecafluoro-1-octanol (6:2 FTOH), PFNA and, perfluorodecanoic acid (PFDA) were purchased from Sigma–Aldrich. Perfluorohexanesulfonic acid (PFHxS) was purchased from LGC standards. The Seahorse XF medium was purchased from Agilent Technologies. The oligomycin, Carbonyl cyanide-p-trifluoromethoxyphenylhydrazone (FCCP), rotenone, antimycin, and fenofibrate were purchased from Sigmar-Aldrich.

**Table 1:** The selection of PFASs tested includes their number of carbon atoms, CAS number, and purity.

PFASs	Carbon atoms	CAS No	Purity (%)
*Perfluorinated carboxylic acid (PFCA)*			
Perfluorobutyric acid (PFBA)	C4	375–22-4	98
Perfluorohexanoic acid (PFHxA)	C6	307–24-4	97
Perfluoroheptanoic acid (PFHpA)	C7	375–85-9	99
Perfluorooctanoic acid (PFOA)	C8	335–67-1	95
Perfluorononanoic acid (PFNA)	C9	375–95-1	97
Perfluorodecanoic acid (PFDA)	C10	335–76-2	98
*Perfluorinated sulfonic acid (PFSA)*			
Perfluorobutanesulfonic acid (PFBS)	C4	375–73-5	97
Perfluorohexanesulfonic acid (PFHxS)	C6	355–46-4	99
Perfluorooctanesulfonic acid (PFOS)	C8	1763–23-1	99*
*Fluorotelomer alcohol (FTOH)*			
2,2,3,4,4,4-Heptafluoro-1-butanol (3:1 FTOH)	C4	382–31-0	95
3,3,4,4,5,5,6,6,6-Nonafluoro-1-hexanol (4:2 FTOH)	C6	2043–47-2	97
3,3,4,4,5,5,6,6,7,7,8,8,8-Tridecafluoro-1-octanol (6:2 FTOH)	C8	647–42-7	97

*PFOS in a concentration of 40% in H_2_O (w/v).

### Cell culture

HepG2 cells were cultured in 75 cm^2^ tissue culture flasks and incubated at 37 ˚C and 5% CO_2_ humidified atmosphere in DMEM supplemented with 10% (v/v) FBS and 100 IU/mL of pen-strep. Once the cell culture reached 80% confluency, it was split. The cell culture medium was refreshed three times a week.

### Cell viability assay

Cell viability of HepG2 cells was measured using MTT as described previously^22^. First, HepG2-cells were seeded in a 96-well culture plate at a cell density of 4 × 10^4^ cells/well and cultured overnight. After 24 h, the culture medium was removed, and cells were washed and subsequently exposed to the PFASs (at 0, 5, 25, 50, 100, 200, 400, and 800 µM) added from stocks in DMSO to the culture medium for a final volume of 200 µL (final DMSO concentration 0.1% (v/v)) for 3 h or 24 h. The selected PFASs that were tested are presented in Table 1. A positive control of 1% (v/v) Triton-X and a vehicle control of 0.1% (v/v) DMSO were also included.

After exposure, the supernatant was discarded, and each well was washed once with DPBS. Then, 200 µL MTT solution (0,5 mg/mL) in DPBS was added to each well and left to rest for 1 h at 37 °C. After this incubation, the supernatant was removed, and the wells were washed with DPBS. Finally, 200 µL DMSO was added to each well, and after 30 min incubation at room temperature, the absorbance was measured at 540 nm with a Synergy HTX Multi-Mode Reader (BioTek, Winooski, USA). Cell viability has been calculated as the mean absorbance of treated samples and has been compared to the vehicle control, which was set at 100%. Three experimental replicates have been performed for each sample, with three biological replicates within each experiment.

### Intracellular ROS formation assay

Intracellular ROS formation in HepG2 cells was measured using DCFH-DA as described previously^22^. HepG2 cells were seeded in 96-well culture plates at a cell density of 4 × 10^4^ cells/well and left to rest overnight. Afterward, the culture medium was discarded, and each well was washed with DPBS. Each well was treated with 100 µL DCFH-DA 10 µM for 40 min. The supernatant was then discarded, and the cells were washed twice with DPBS. Subsequently, the cells were exposed to 200 µL PFAS solution in phenol red free DMEM. Specific concentrations and exposure times are presented in the results section. In addition to the PFASs treatments, a positive control of 1 mM H_2_O_2_ and a vehicle control of 0.1% (v/v) DMSO were included. At the end of the incubation time, the relative fluorescence unit (RFU) of DCF was measured using a Synergy HTX Multi-Mode Reader (BioTek, Winooski, USA) with an excitation wavelength of 500 nm and emission wavelength of 540 nm. The increase in ROS generation was then calculated using the ratio of the RFU between treated samples and vehicle control. Three experimental replicates have been performed for each sample, with three biological replicates within each experiment.

### Protein quantification assay

Protein quantification was performed using the BCA protein assay kit from Thermofisher using the provider’s protocol each time the protein content was required. Prior to this assay, the cells seeded in a 96-well plate were lysed using Pierce lysis buffer. 50 µL were added to each well and incubated on ice for 15 min. Afterward, 10 µL of each sample was used for Protein quantification.

### Lipid peroxidation assay

Lipid peroxidation was measured using a thiobarbituric acid-reactive substance (TBARS) assay following the protocol outlined by Potter et al. (2011)^23^. HepG2 were seeded at a density of 1.5 × 10^6^ cells per well in 6-well plates and left to incubate overnight. Each well was treated with either PFOA, PFOS, or 6:2 FTOH at a concentration of 50, 100, or 200 µM for 3 or 24 h. Positive controls consisted of 5 mM diethyl malonate (DEM) and a negative control of cell culture DMEM.

After exposure, the supernatant was collected and centrifuged at 2,800 g for 15 min to eliminate cellular debris. Meanwhile, the cells were washed twice using ice-cold DPBS and lysed in 200 ml RIPA buffer. The cell lysate was collected and centrifuged at 2,800 g for 15 min to eliminate cell debris. Then, it was stored for protein quantification.

A calibration curve was prepared by diluting 3,4-Methylenedioxyamphetamine (MDA) in ice-cold water at a concentration of 0.007, 0.015, 0.031, 0.063, 0.125, 0.25, 0.5, 1, and 4 nmol/L. A quality control consisting of MDA at 0.8 nmol/L in water was prepared in addition to the controls.

400 µL TCA 15% (v/v) and 800 µL of a solution composed of TBA 0.67% (v/v) and BHT 0.01% (v/v) were added to 500 µL of either supernatant, calibration curve, or QC. Each sample was vortexed and heated for 20 min in a 95 °C water bath. Afterward, the samples were cooled down, and 3 ml butanol was added and mixed using inversion. The samples were centrifuged for 5 min at 2,800 g for 10 min, and 200 µL of the butanol supernatant was transferred to a 96-well plate. The plate was black with a transparent bottom for measurements.

The RFU was measured with an excitation wavelength of 530 nm and an emission wavelength of 550 nm. The final concentration of TBARS for each condition was calculated in nmol/L and corrected for the total protein content in each well.

### Lysosomal leakage assay

Permeabilization of the lysosome membranes was measured using acridine orange change in fluorescence following the uptake of this dye in the lysosomes. HepG2 cells were seeded in 96-well culture plates at 4 × 104 cells/well cell density and left to rest overnight. The supernatant was discarded, and 100 µL acridin orange at 2 µg/ml was added and left for 10 min. After incubation, the supernatant was discarded, and each well was washed twice with DPBS. Subsequently, the cells were exposed to 200 µL PFAS solution in phenol red-free DMEM. Specific concentrations and exposure times are presented in the results section.

After exposure, the RFU of acridine orange was measured at an excitation wavelength of 495 nm and an emission wavelength of 538 nm. Afterward, the supernatant was discarded, and each well was washed twice with DPBS. The cells were then lysed with 50 µL RIPA buffer on ice for 15 min before measuring the protein content using the protein quantification assay. The overall acridine orange fluorescence was corrected for the total protein content in each well. The lysosomal leakage was calculated as the RFU ratio between the treated sample and vehicle control. Three experimental replicates have been performed for each sample, with three biological replicates within each experiment.

### Peroxisome purification

HepG2 cells were cultured in T75 flasks until reaching 80% confluency. Cells were detached using 2 ml trypsin per flask and incubated for 3 min. The trypsinization was stopped using 3 ml of cell culture medium, and the cell suspension was subsequently centrifuged for 5 min at 250 g. The supernatant was discarded, and the cells were washed by resuspending them in 10 ml ice-cold DPS before centrifuging them again for 5 min at 250 g. The washing was repeated before resuspending the pellet in a 5 ml homogenization medium. The cells were lysed using 50 strokes with a Dounce homogenizer. The cell lysate was centrifuged at 2,800 g for 15 min at 4 °C to eliminate cellular debris. The homogenate was then collected and kept on ice.

The light mitochondrial pellet was then first purified. An ultracentrifuge tube was prepared by layering 2 ml Percol 100%, then 6 ml Percol 40% (v/v), sucrose 0.25 M, and finally 5 ml cell homogenate. The tubes were centrifuged for 30 min at 50,000 g at 4 °C, and the top band was collected.

The peroxisome fraction was collected by mixing the light mitochondrial pellet with Percol 40% (v/v) and 0.25 M sucrose to a final volume of 12.5 ml. The solution was placed in an ultracentrifuge tube and centrifuged for 30 min at 60,000 g at 4°C. The lowest band was collected and reconstituted in a homogenization medium to a final volume of 2 ml.

### Catalase activity assay

The protein content of the purified peroxisome was measured, and the solution was diluted to a final concentration of 250 ng/ml. 25 µL of diluted peroxisome was mixed with 75 µL of hydrogen peroxide 125 µM reaction buffer and Triton-X 0.01% (v/v) and aliquoted to a black plate with a clear bottom. The solution was incubated for 5 min before adding 5 µL 3-Amino-1,2,4-triazole 1mM. After adding 95 µL of a revelation buffer composed of DCF 10 µM, horseradish peroxidase was added and incubated for 15 min.

At the end of the incubation time, the RFU of DCF was measured using a Synergy HTX Multi-Mode Reader (BioTek, Winooski, USA) with an excitation wavelength of 500 nm and emission wavelength of 540 nm. The catalase activity was expressed as the ratio of the samples to a negative control consisting of a homogenization buffer. Three experimental replicates have been performed for each sample, with three biological replicates within each experiment.

### ACOX1 activity assay

The protein content of the purified peroxisome was measured, and the solution was diluted to a final concentration of 250 ng/ml. 25 µL of diluted peroxisome was mixed with 75 µL of reaction buffer composed of palmitoyl-CoA 75 µM and Triton-X 0.01% (v/v) and aliquoted to a black plate with a clear bottom. The samples were incubated for 15 min before adding 100 µL of a revelation solution composed of 3-Amino-1,2,4-triazole 50 µL and DCF (50 µM) and incubated for 15 min.

At the end of the incubation time, the RFU of DCF was measured using a Synergy HTX Multi-Mode Reader (BioTek, Winooski, USA) with an excitation wavelength of 500 nm and emission wavelength of 540 nm. ACOX1 activity was expressed as the ratio between the samples to a vehicle control of cells exposed to DMSO 0.1% (v/v). To confirm an increase in ACOX1 activity, a positive control consisting of purified peroxisome from cells exposed to fenofibrate (40 µM) was employed. Three experimental replicates have been performed for each sample, with three biological replicates within each experiment.

### Seahorse assay

Mitochondrial functioning after PFASs exposure has been measured using the Seahorse assay from Agilent technologies. The assay was performed following the manufacturer’s protocol. Cells were seeded in a seahorse XF 96-well plate at a density of 3.29 × 10^3^ cells per well and left to rest overnight. The supernatant was discarded, and the cells were washed once with DPBS. Subsequently, the cells were exposed to 100 µL PFAS solution in cell culture medium. Specific concentrations and exposure times are presented in the results section. A negative control comprised cells exposed to DMSO 0.2% (v/v).

After 24 h of exposure, the supernatant was discarded, each well was washed once with DPBS, and 175 µL XF media was added to each well. The plate was incubated for 1 h at 37 °C in a CO_2_-free incubator. At the end of the incubation time, the mitochondrial function was measured through the oxygen consumption rate (pmol/min). The first injection consisted of 8 µM oligomycin, the second injection of 9 µM FCCP, and the third injection of 1 µM rotenone/antimycin. Each injection consisted of 25 µL. The overall oxygen consumption rate was measured after each injection. Mitochondrial ATP production was calculated as the basal oxygen consumption rate minus the oxygen consumption rate after oligomycin injection. Mitochondrial spare capacity was measured by the oxygen consumption rate minus the baseline oxygen consumption rate after the FCCP injection. Proton leak was measured using the oxygen consumption rate after the oligomycin injection minus the oxygen consumption rate after the rotenone/antimycin injection. The total mitochondrial oxygen consumption rate was measured after the FCCP injection minus the oxygen consumption rate after the rotenone/antimycin injection.

After the oxygen consumption measurement, the supernatant was discarded, the cells were lysed using SDS 0.01% for 30 min, and a protein concentration assay was performed to correct the oxygen consumption values.

### Antioxidant treatment

Antioxidant treatment was performed 24 h prior to the particular assays. HepG2 cells were exposed to 200 µL antioxidant solution in phenol red-free DMEM. The tested antioxidants included 40 µM quercetin or 10 µM mito-tempo. After exposure, the supernatant was discarded, and each well was washed three times with DPBS, and the plate was immediately used for the subsequent assay.

### Statistical analysis

Data were analyzed using GraphPad Prism software (v. 5.00, GraphPad Software, San Diego, CA, USA). Statistical significance of changes in cell viability, reactive oxygen species generation, lipid peroxidation, lysosomal leakage, seahorse assay, catalase activity, and ACOX1 activity values due to PFAS exposure compared to the vehicle control cohort was calculated by Student’s t-test and is indicated as following: * = p < 0.05.

## Results

### Cell viability assay

To determine the role of ROS generation by PFASs in their ability to reduce cell viability, HepG2 cells have been treated with either quercetin or mito-tempo before exposure to PFASs. Cell viability was assessed using MTT, and cell viability was expressed as the ratio between the test condition and the negative control.

**Fig. 1:**
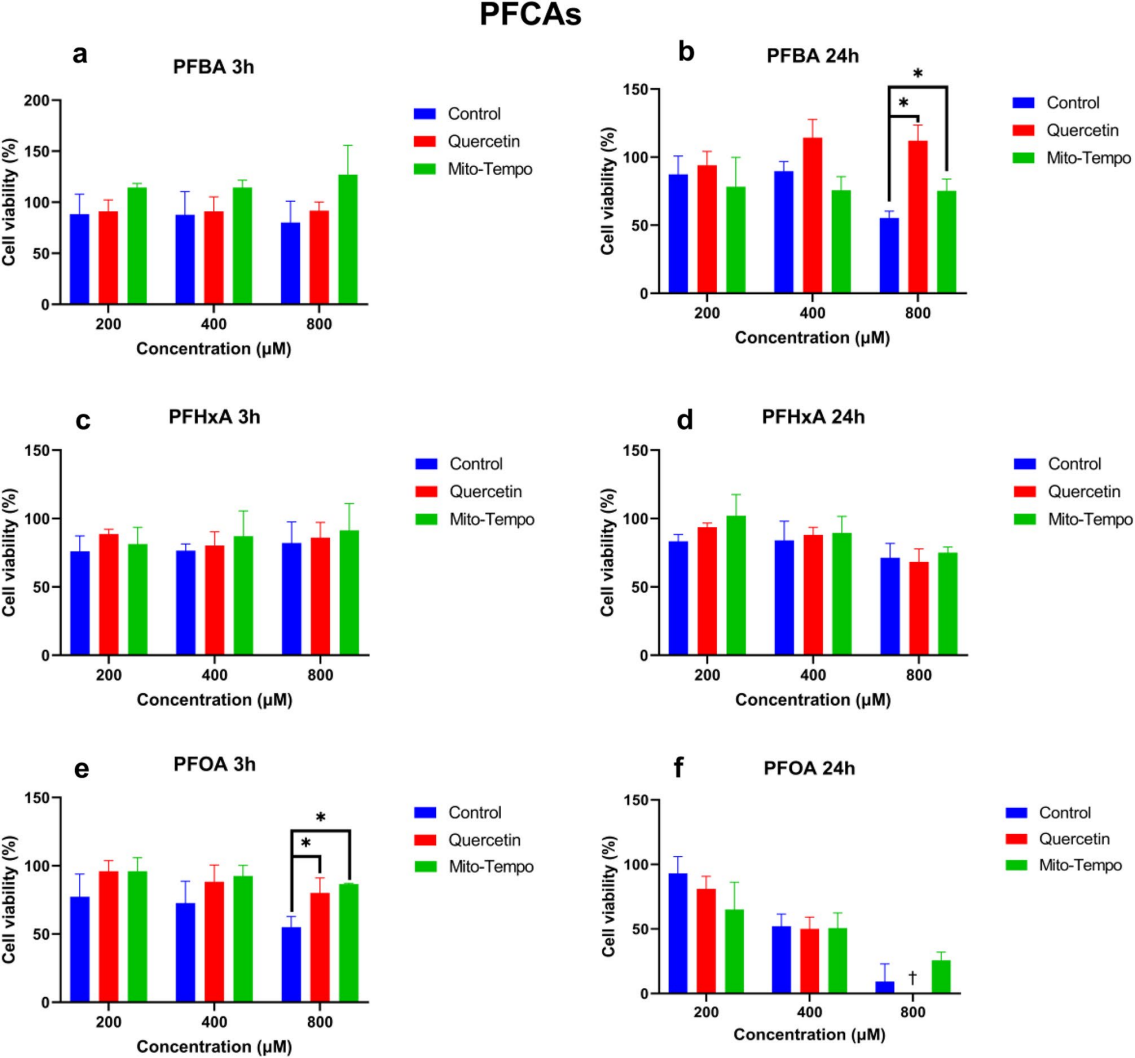
The effect of different PFCAs on HepG2 cell viability with and without antioxidant treatment. Antioxidant exposure time was 24 h, followed by a PFAS exposure of either 3 or 24 h. For each condition, a control consisting of PFAS without antioxidants was tested. The Y-axis corresponds to the cell viability in %, where the viability of untreated (solvent control) HepG2 cells was set at 100%. The X-axis shows the PFCAs in 200, 400, and 800 µM concentrations. Figures a, c, and e, while b, d, and f show the results for PFBA, PFHxA, and PFOA at 3 h and 24 h, respectively. Each condition was tested in triplicate. Data are expressed as mean ± SD. *= p < 0.05. † = below limit of detection. To determine whether exposure to antioxidants significantly increased cell viability, antioxidant-treated samples were compared to PFAS-only samples.

The effects of PFCA exposure with and without pre-exposure to the antioxidants quercetin and mito-tempo on HepG2 cell viability are presented in Fig. 1. Most PFCAs concentration-dependently decreased cell viability after 3 and 24 h of exposure.

After 3 h exposure, a decrease in cell viability was observed for PFOA, while no effect was observed for PFBA and PFHxA. Pre-treatment with quercetin did not significantly improve cell viability after 3 h of PFCAs exposure, except for PFOA at 800 µM (P = 0.0146). In general, pre-treatment with an antioxidant appears to increase cell viability.

After 24 h, reduced cell viability was observed for PFBA, PFHxA, and PFOA. Pre-treatment with quercetin significantly increased cell viability for PFBA at 800 µM (P = 0.018). After 24 h, mito-tempo only marginally improved cell viability for PFOA and significantly increased it for PFBA at 800 µM (P = 0.023). P-values for each condition are presented in supplementary material table S1.

**Fig. 2:**
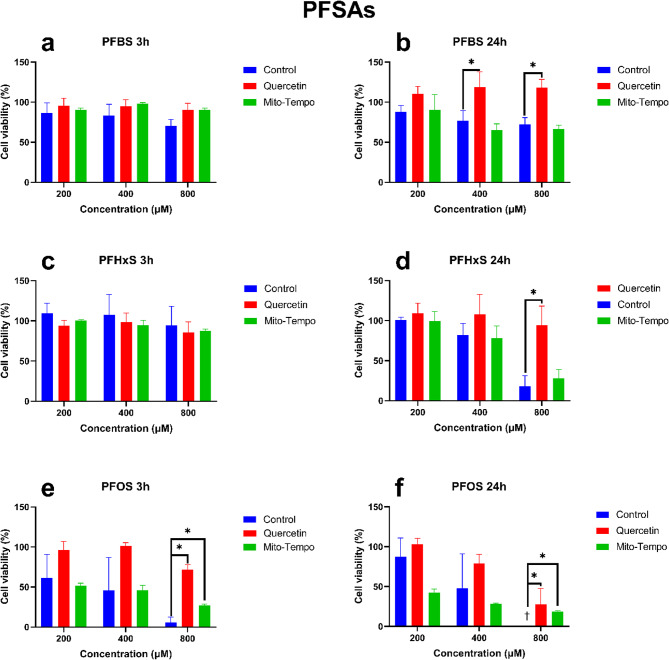
The effect of the different PFSAs on HepG2 cell viability with and without antioxidant treatment. Antioxidant exposure time was 24 h, followed by a PFAS exposure of either 3 or 24 h. For each condition, a control consisting of PFAS without antioxidants was tested. The Y-axis corresponds to the cell viability in %, where the viability of untreated (solvent control) HepG2 cells was set at 100%. The X-axis shows the PFSAs in 200, 400, and 800 µM concentrations. Figures a, c, and e, while b, d, and f show the results for PFBS, PFHxS, and PFOS at 3 h and 24 h, respectively. Each condition was tested in triplicate. Data are expressed as mean ± SD. *= p < 0.05. † = below limit of detection. To determine whether exposure to antioxidants significantly increased cell viability, antioxidant-treated samples were compared to PFAS-only samples.

The effects of PFSA exposure with and without pre-exposure to the antioxidants quercetin and mito-tempo on HepG2 cell viability are presented in Fig. 2. In general, all PFSAs concentration-dependently decreased cell viability after 3 and 24 h of exposure.

After 3 h of exposure, a decrease in cell viability was observed for PFBS, PFHxS, and PFOS. Pre-treatment with quercetin significantly improves cell viability after 3 h exposure to PFOS at 800 µM (P = 0.010). Similarly, after 3 h, mito-tempo significantly improved cell viability for PFOS at 800 µM (P = 0.038) (Figs. 2a, c and e). Generally, pre-treatment with quercetin increased cell viability, while mito-tempo had a lower effectiveness.

After 24 h, reduced cell viability was observed for PFBS, PFHxS, and PFOS. Pre-treatment with quercetin significantly increased cell viability for PFBS, PFHxS, and PFOS. For PFBS, a significant increase was observed at 200 µM (P = 0.003) and 800 MµM (P = 0.041). For PFHxs, a significant increase was observed at 800 µM (P = 0.014). However, mito-tempo only increased cell viability for PFOS at 800 µM (P = 0.038). P-values for each condition are presented in the supplementary material Table S1.

### Reactive oxygen species generation assay

To the determine potential origin of ROS generation by PFASs, HepG2 cells have been treated with either quercetin or mito-tempo before exposure to PFASs. ROS generation was assessed using DCFHda, and cell viability was expressed as the ratio between the test condition and the negative control.

**Fig. 3:**
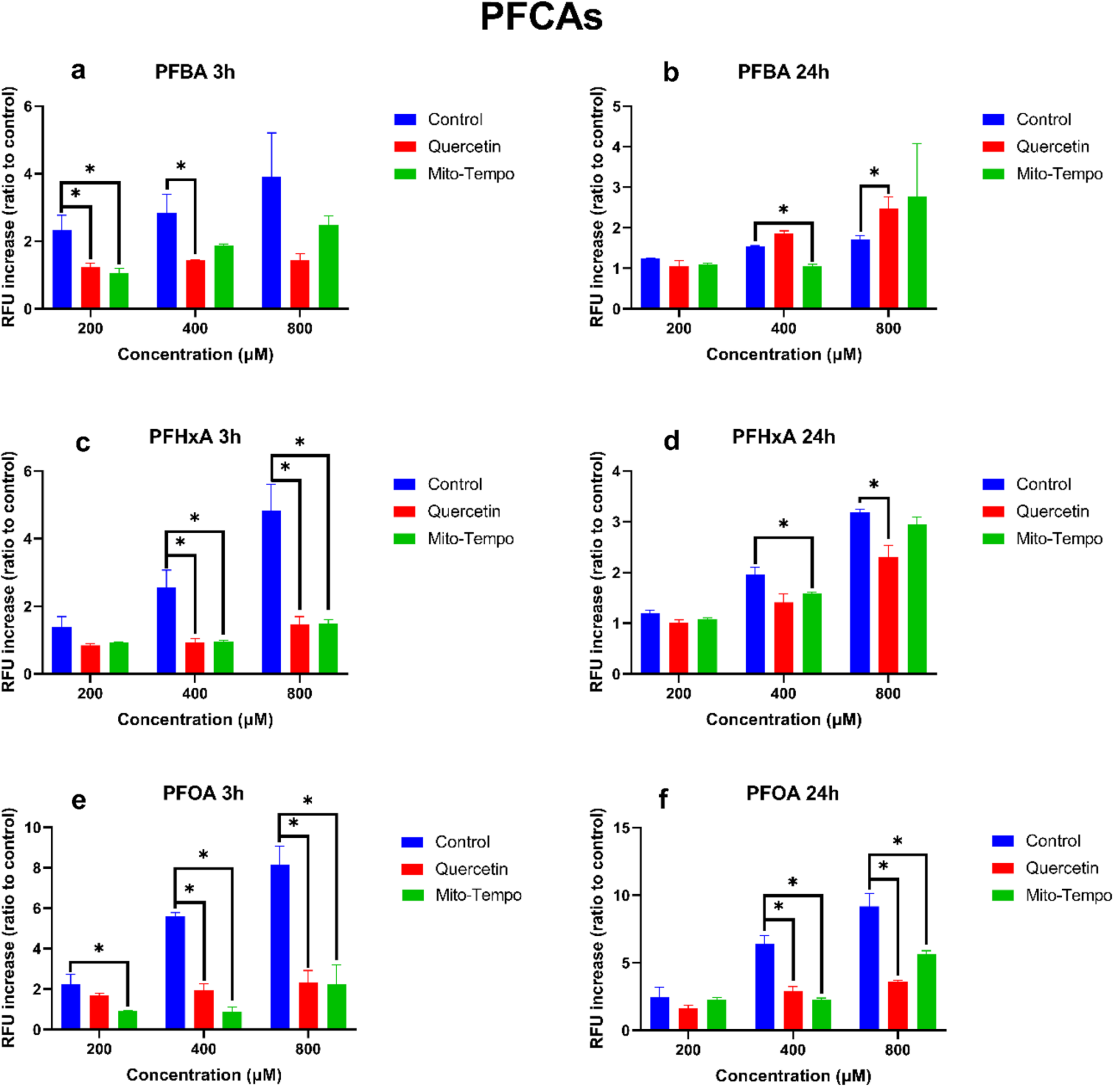
The effect of the different PFCAs on the formation of intracellular ROS in HepG2 with and without antioxidant treatment. Antioxidant exposure time was 24 h, followed by a PFAS exposure of either 3 or 24 h. Figures a, c, and e, while figures b, d, and f present the impact of PFBA, PFHxA, and PFOA after 3 and 24 h, respectively. The Y-axis represents the relative fluorescence increase compared to the negative control. Each condition was tested in triplicate. Data are expressed as mean ± SD. *= p < 0.05. To determine whether exposure to antioxidants significantly reduce ROS generation, antioxidant-treated samples were compared to PFAS-only samples.

The influence of quercetin and mito-tempo on PFCA-induced ROS generation in HepG2 cells is presented in Fig. 3. All PFCAs induced a concentration-dependent increase in ROS formation after 3 and 24 h. After 3 h, quercetin almost completely decreased ROS formation in HepG2 cells exposed to PFBA, PFHxA, and PFOA. For PFBA, a significant decrease was observed at 200 µM (P = 0.036) and 400 µM (P = 0.05). For PFHxA, a significant decrease was observed at 400 µM (P = 0.044) and 800 µM (P = 0.024). A significant decrease was observed for PFOA at 400 µM (P = 0.001) and 800 µM (P = 0.018). After 24 h, a significant decrease in ROS formation by quercetin can be seen for PFBA at 400 µM (P = 0.018), PFHxA at 800 µM (P = 0.028), and PFOA at 400 µM (P = 0.005) and 800 µM (P = 0.008).

Mito-tempo significantly decreased ROS generation in HepG2 cells exposed to PFHxA, PFOA, and PFBA for 3 h. For PFBA, a significant decrease was observed at 200 µM (P = 0.05). For PFHxA, a significant decrease was observed at 400 µM (P = 0.028) and 800 µM (P = 0.014). After 24 h, a significant decrease in ROS generation by mito-tempo was observed only for PFBA, PFHxA, and PFOA. For PFBA, a significant decrease was measured at 400 µM (P = 0.001). For PFHxA, a significant decrease was measured at 400 µM (P = 0.036). A significant decrease is measured for PFOA at 400 µM and 800 µM. P-values for each condition are presented in supplementary material table S1.

**Fig. 4:**
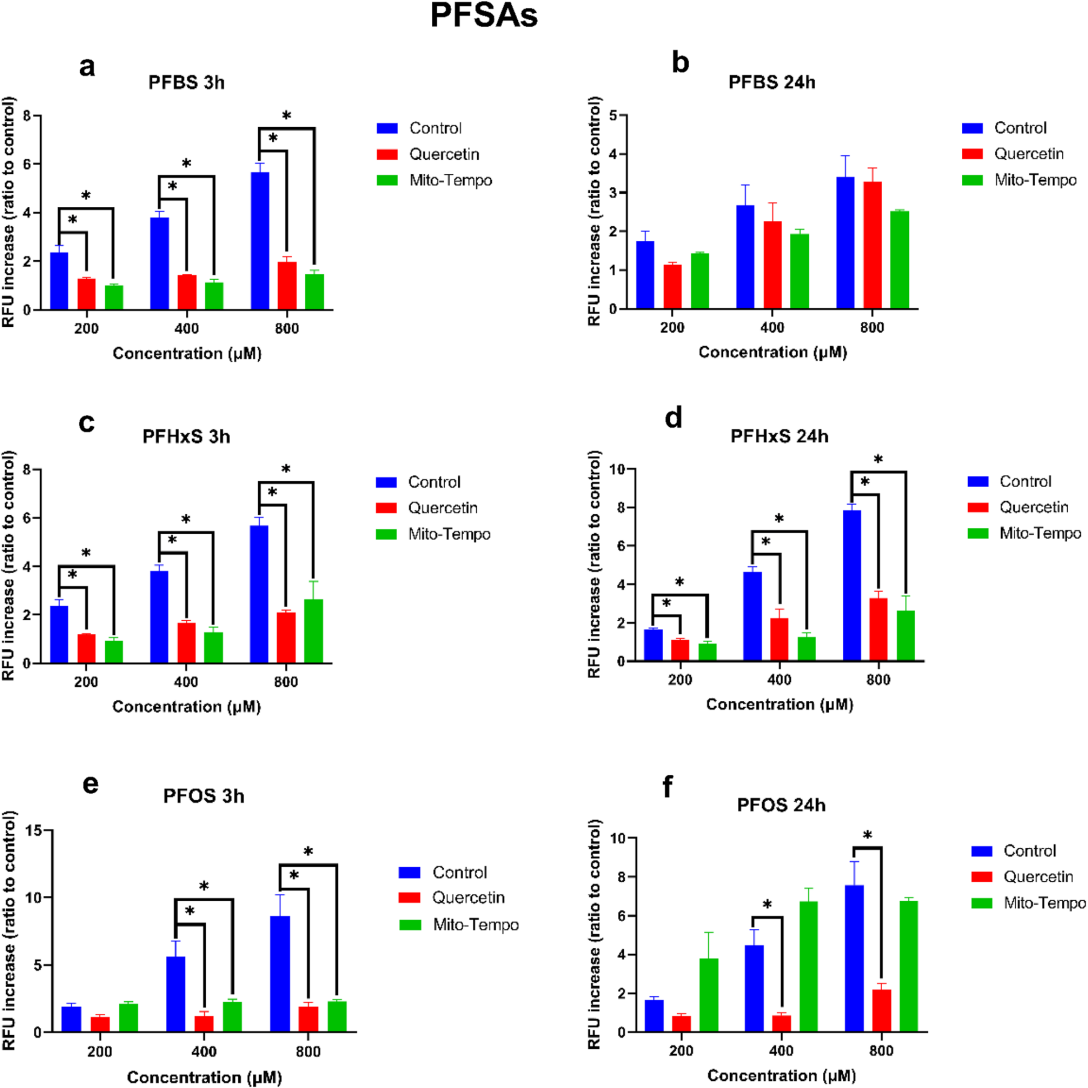
The effect of the different PFSAs on the formation of intracellular ROS in HepG2 with and without antioxidant treatment. Exposure time for antioxidants was 24 h, followed by a PFAS exposure of either 3 or 24 h. Figures a, c, and e, while b, d, and f present the impact of PFBS, PFHxA, and PFOS after 3 and 24 h, respectively. The Y-axis represents the relative fluorescence increase compared to the negative control. Each condition was tested in triplicate. Data are expressed as mean ± SD. *= p < 0.05. To determine whether exposure to antioxidants significantly reduce ROS generation, antioxidant-treated samples were compared to PFAS-only samples.

The influence of quercetin and mito-tempo on PFSA-induced ROS generation in HepG2 cells is presented in Fig. 4. All PFSAs induced a concentration-dependent ROS formation increase after 3 and 24 h. Quercetin significantly decreased ROS generation in HepG2 cells exposed to PFBS, PFHxS, and PFOS for 3 h. For PFBS, a significant decrease is measured at 200 µM (P = 0.025), 400 µM (P = 0.005), and 800 µM (P = 0.008). For PFHxS, a significant decrease is measured at 200 µM (P = 0.02), 400 µM (P = 0.009), and 800 µM (P = 0.006). For PFOS, a significant increase is measured at 400 µM (P = 0.028), and 800 µM (P = 0.001). After 24 h, a significant decrease in ROS formation by quercetin was observed for PFHxS and PFOS. For PFHxS, a significant decrease is measured at 200 µM (P = 0.018), 400 µM (P = 0.007), and 800 µM (P = 0.005). For PFOS, a significant decrease is measured at 800 µM (P = 0.03).

Mito-tempo significantly decreased ROS generation in HepG2 cells exposed to PFBS, PFHxS, and PFOS for 3 h. For PFBS, a significant decrease was observed at 200 µM (P = 0.016), 400 µM (P = 0.005), and 800 µM (P = 0.005). For PFHxS, a significant decrease is measured at 200 µM (P = 0.023), 400 µM (P = 0.011), and 800 µM (P = 0.009). For PFOS, a significant increase is measured at 800 µM (P = 0.038). After 24 h of exposure, mito-tempo significantly decreased ROS generation by PFOS. For PFHxS, a significant decrease was observed at 200 µM (P = 0.005), 400 µM (P = 0.003), and 800 µM (P = 0.013). For PFOS, a significant decrease was observed at 800 µM (P = 0.038). P-values for each condition are presented in supplementary material table S1.

### Lysosomal leakage assay

To determine whether lysosome leakage is involved in PFAS-induced ROS generation, HepG2 cells have been treated with acridine orange before exposure to PFASs. The permeabilization of the membrane was then measured as the fluorescence shift of acridine orange. Furthermore, the effect of antioxidants on lysosomal leakage has been assessed using quercetin and mito-tempo.

First, permeabilization of the lysosome membrane by PFASs has been investigated by exposing HepG2 cells to PFASs for 3 h. Each condition is compared to a vehicle control to determine whether exposure to antioxidants significantly reduced lysosomal leakage.

**Fig. 5:**
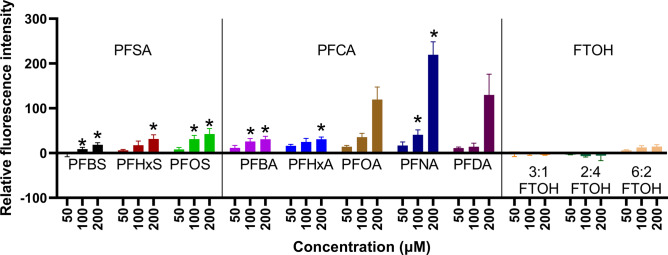
The effect of the different PFASs on HepG2 lysosomal membrane after 3 h of exposure. The Y-axis represents the relative fluorescence increase compared to the negative control. Each condition was tested in triplicate. Data are expressed as mean ± SD. *= p < 0.05. Statistical significance was established in comparison to the vehicle control.

**Fig. 6:**
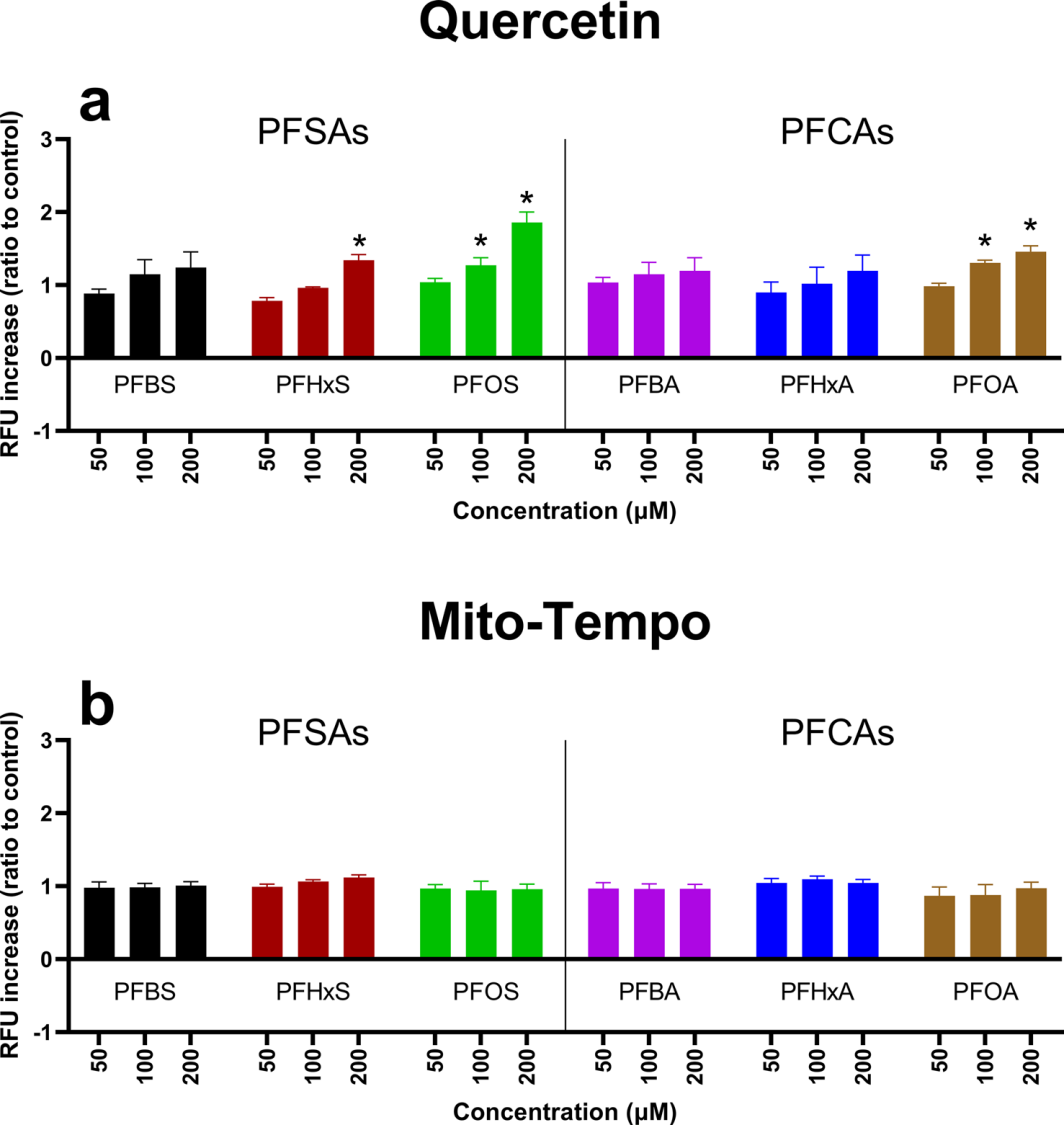
The effect of 24 h pre-exposure to quercetin or mito-tempo on HepG2 lysosomal membrane permeabilization by PFASs after 3 h of exposure. The Y-axis represents the relative fluorescence increase compared to the negative control treated with DMSO 0.2% (v/v). Each condition was tested in triplicate. Data are expressed as mean ± SD. *= p < 0.05. Statistical significance was established in comparison to the vehicle control.

The effects of PFASs on lysosomal membrane integrity are presented in Figs. 5 and 6. Lysosomal leakage significantly increased after concentration-dependent exposure to all PFCAs and PFSAs. No significant lysosomal leakage was observed at any tested concentrations for FTOH. To further test whether the permeabilization of the lysosome membrane is caused by PFASs directly or ROS generated by PFASs, the experiment was repeated after pre-treatment of the cells by either quercetin or mito-tempo. Pre-treatment with quercetin greatly decreased lysosomal leaking; however, a concentration-dependent increase can still be observed for PFHxS, PFOS, and PFOA. Pre-treatment with Mito-Tempo greatly decreased lysosomal leakage for PFBS, PFHxS, PFOS, PFBA, PFHxA, and PFOA with no apparent concentration-dependent increase. P-values for each condition are presented in supplementary material table S1.

### Lipid peroxidation assay

To investigate lipid peroxidation as a potential source of ROS generation, HepG2 cells have been exposed to either PFOA, PFOS, or 6:2 FTOH before being lysed, and the amount of TBARS has been measured. As a chain-length effect of PFASs on ROS generation has been previously established, only PFASs with eight carbons have been tested to investigate the effect of the headgroup.

**Fig. 7:**
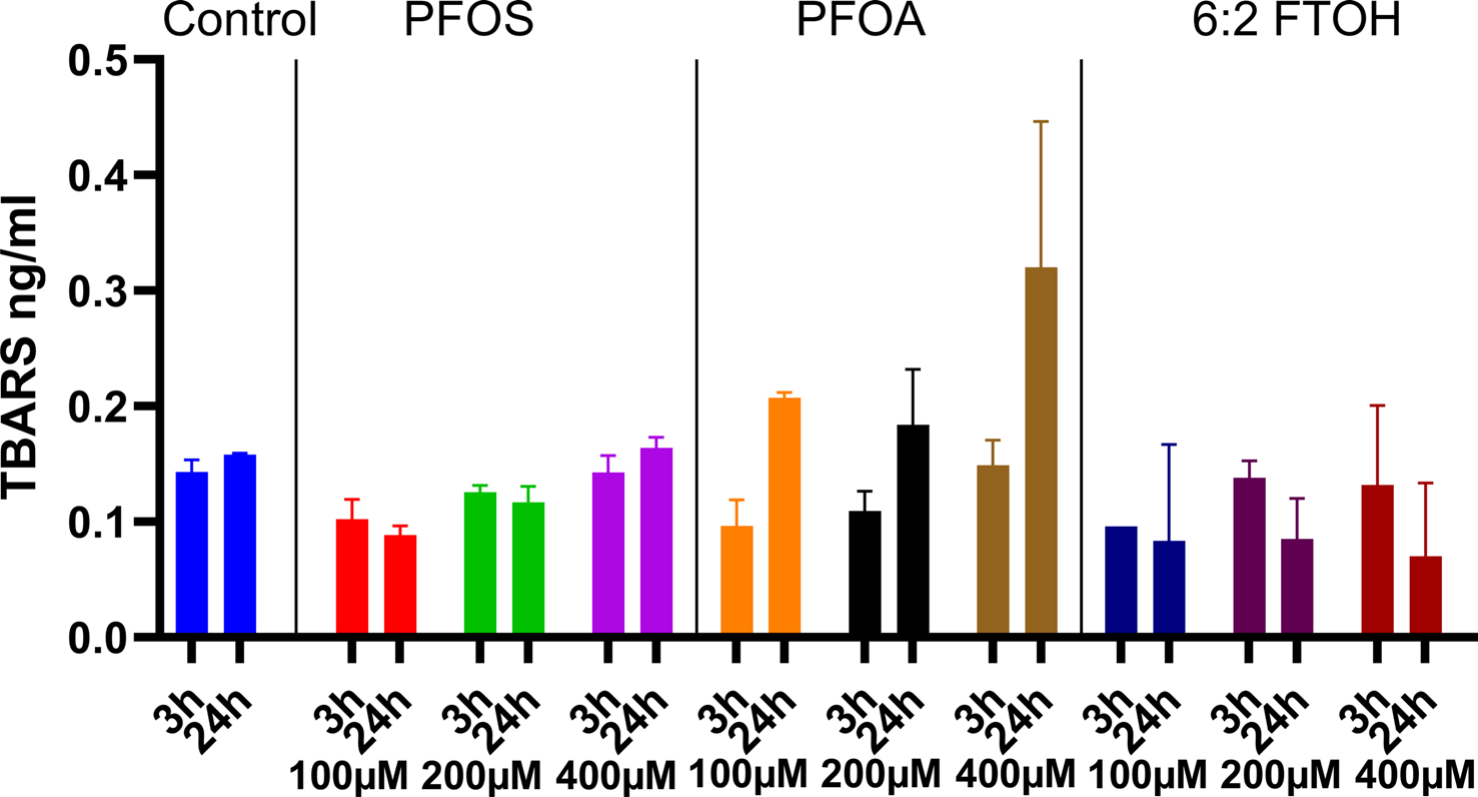
The effect of the different PFASs on HepG2 lipid peroxidation after 3 h exposure. The Y-axis represents measured TBARS in ng/ml. Each condition was tested in triplicate. Data are expressed as mean ± SD. Statistical significance was established in comparison to the vehicle control.

The effects of PFASs on lipid peroxidation are presented in Fig. 7. No significant increase in TBARS was measured for PFOA, PFOS, or 6:2 FTOH at the tested concentrations and time points. After 24 h of exposure, an increase in TBARS is measured for PFOA at 100, 200, and 400, although non-significant.

### Seahorse assay

To investigate the role of mitochondrial dysfunction as a potential source of ROS generation, HepG2 cells have been exposed to either PFOA, PFOS, or 6:2 FTOH, and the oxygen consumption rate has been measured.

**Fig. 8:**
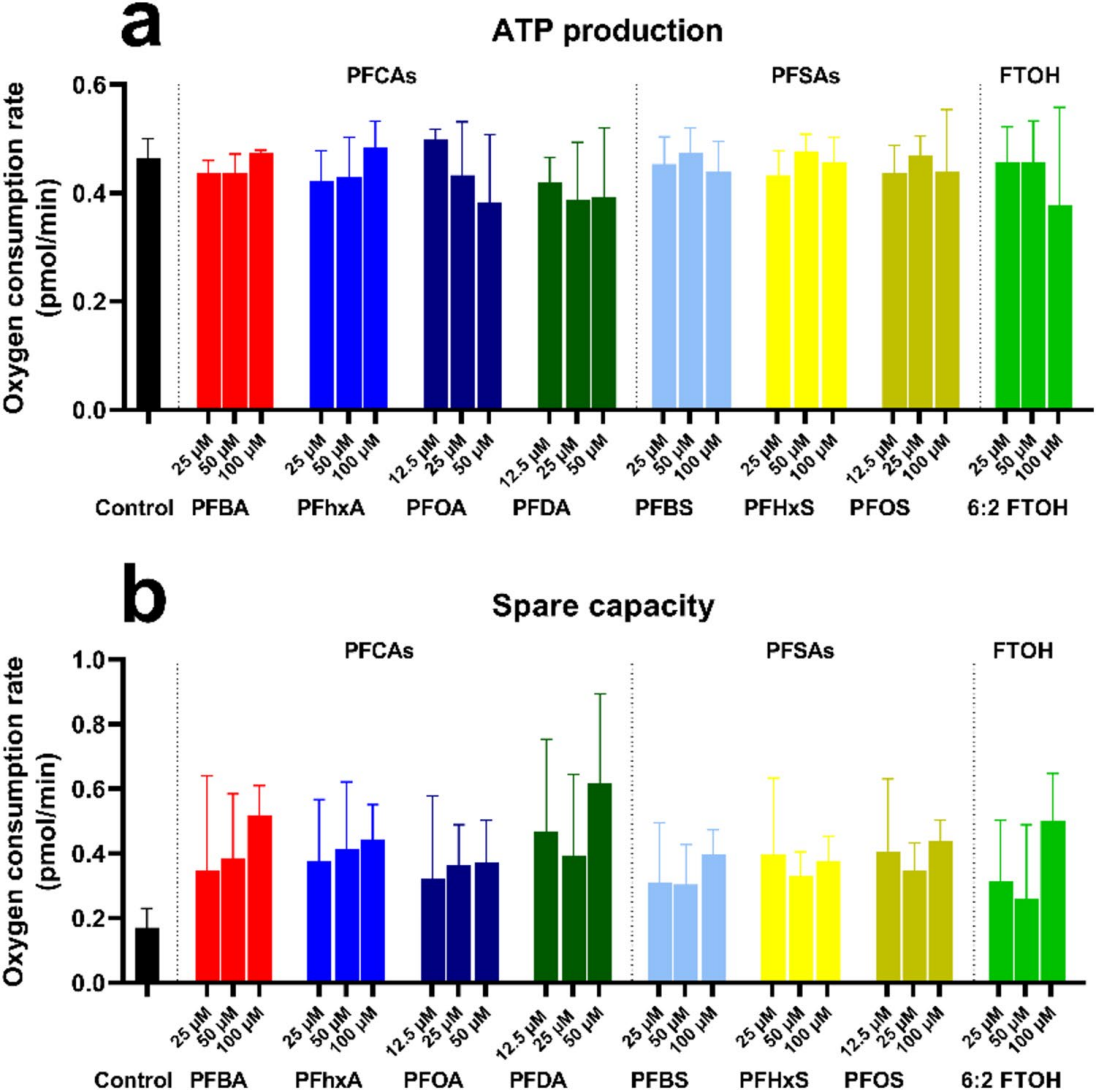
The effect of the different PFASs on HepG2 mitochondrial functioning after 3 h of exposure. Mitochondrial ATP production (a), and overall mitochondrial respiration (b) are shown. The Y-axis represents the oxygen consumption rate (pmol/min). Each condition was tested in triplicate. Data are expressed as mean ± SD. Statistical significance was established in comparison to the vehicle control.

The effect of PFCAs, PFSAs, and FTOHs on mitochondrial activity was assessed using the Seahorse assay and is presented in Fig. 8. An increase in mitochondrial respiration was observed for PFBA, PFHxA, PFOA, PFDA, PFBS, PFHxS, and PFOS, although not significant (Fig. 8a). In addition, PFCAs and PFSAs increased the mitochondrial spare capacity of the HepG2 cells (Fig. 8b). Exposure to PFASs did not appear to significantly impact mitochondrial ATP production and proton leakage at any tested concentration (Figure S1).

### Peroxisomal function assay

HepG2 cells were exposed to either PFOA, PFOS, or 6:2 FTOH before being lysed, and the peroxisome fraction was collected to investigate peroxisomal dysfunction as a potential source of ROS generation. As a chain-length effect of PFASs on ROS generation has been previously established, only PFASs with eight carbons have been tested to investigate the eventual headgroup effect. To determine whether exposure to PFASs disrupts peroxisomal function, PFAS-treated samples were compared to vehicle control samples.

**Fig. 9:**
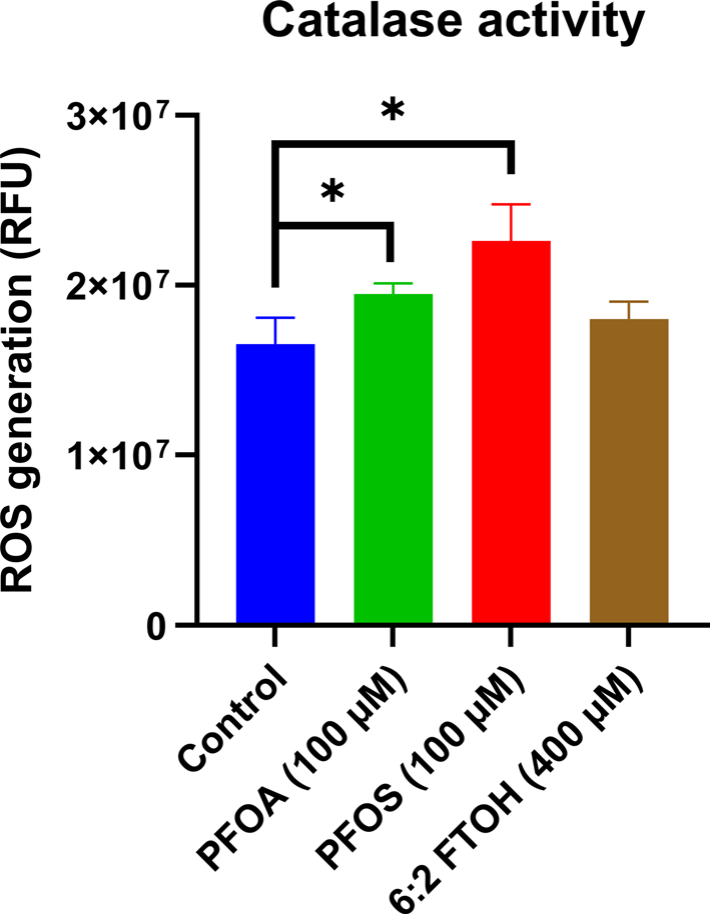
The effect of different PFASs on H_2_O_2_ degradation by peroxisomal catalase after 24 h exposure. The control condition corresponds to cell culture medium with 0.2% DMSO. The Y-axis corresponds to the measured relative fluorescence intensity. Each condition was tested in triplicate. Data are expressed as mean ± SD. *= p < 0.05. Statistical significance was established in comparison to the vehicle control.

The effect of PFOA, PFOS, and 6:2 FTOH on peroxisomal catalase degradation of H_2_O_2_ was measured using the DCFH assay and is presented in Fig. 9. An increase in relative fluorescence indicates a decreased catalase activity. A significant increase was observed for 100 µM of PFOA (P = 0.038) and PFOS (P = 0.017) but not for 400 µM 6:2 FTOH.

The effect of PFOA, PFOS, and 6:2 FTOH on peroxisomal β-oxidation was measured after adding palmitoyl-CoA. No significant increase was observed after exposure to PFOA (100 µM), PFOS (100 µM), or 6:2 FTOH (Figure S2).

## Discussion

Since PFASs originally have become a source of concern for human safety, their hepatotoxicity has been established both in vitro and in vivo. PFOA and PFOS have been shown to cause hepatotoxicity in mice, rats, monkeys, and humans^24–26^. Similarly, the plasma concentrations of legacy PFASs such as PFOA and PFOS have been linked to human hepatic hypertrophy and fibrosis^27^. However, since for the majority of PFASs it is unknown if they exert hepatoxic effects and the mechanisms of toxicity are lacking, the current study aims to provide further insights into the mechanism of action by assessing the effect of a selection of PFASs on a sub-cellular level in hepatocytes. Various in vivo and in vitro studies have linked the cytotoxic effects of PFASs to their ability to induce ROS generation^22,28–30^. However, little is available on the origin of the ROS caused by PFASs exposure, nor on the key molecular event. As such, the present study has investigated the potential cytosolic or mitochondrial origin for ROS generation caused by PFASs exposure.

### Source of ROS

The potential sub-cellular site or sites of ROS generation has been assessed using two antioxidants: a generic antioxidant, quercetin, and a mitochondrial-targeted antioxidant, mito-tempo^33,34^, allowing us to determine if the initial site of ROS generation might be the cytosol, the mitochondria, or both^33,35^. As expected, pre-treating HepG2 cells with antioxidants significantly reduced PFAS-induced ROS generation after 3 and 24 h of exposure (Figs. 1 and 2). After 3 h of exposure to PFCAs, ROS generation was nearly completely remediated by quercetin treatment. Similar results were observed for PFSAs. The effectiveness of quercetin appeared to drop after 24 h, but it still remediated some of the ROS generation, except for PFBS. Unlike other PFASs, no significant difference could be observed between quercetin-treated and untreated cells after PFBS exposure, indicating a potential difference in the mechanism of action leading to ROS generation.

Treatment with mito-tempo led to a similar effect to quercetin for PFCAs (Figs. 1 and 2). A significant decrease was observed after 3 h, nearly completely remediating ROS generation except for the highest concentrations. Similarly, after 24 h, the ROS generation was significantly reduced but was not completely remediated. These results indicate that ROS generation by PFCAs is partly tied to the mitochondrial compartment. While PFSAs behaved similarly after 3 h, only ROS generation caused by FBS exposure was reduced after 24 h. The present data confirm that after a 3-h exposure, the mitochondria are the potential key site of ROS generation for both PFCAs and PFSAs. However, after 24 h following PFSAs exposure, another source for ROS generation superseded that of the mitochondria. As such, while ROS appeared to be the cause of the reduced cell viability after acute exposure, the origin of said ROS depended on the tested compounds. Indeed, the ROS generation by PFCAs could be remediated by mito-tempo, indicating that the mitochondrial compartment plays an important role. The same effect was not observed for PFSAs. Therefore, it can be concluded that PFCAs lead to greater mitochondrial dysfunction than PFSAs. However, it is not yet possible to determine if it is due to a difference in affinity or multiple mechanisms of action leading to ROS generation.

### ROS as the cause of cell death

Our study also investigated the relationship between ROS formation and cytotoxicity. For PFCAs, pre-treatment with quercetin significantly improved cell viability for PFOA (Figs. 3 and 4). However, due to the lower toxicity of PFBA and PFHxA for HepG2, no clear improvement in cell viability could be observed^27^. After 24 h, quercetin only significantly improved cell viability after PFBA exposure. Similar results were observed for PFSAs after 3 h of exposure, with significant improvement in cell viability after PFOS exposure. However, unlike for PFCAs, quercetin improved cell viability for all tested PFSAs after 24 h.

Similar results were observed for the mitochondrial-specific antioxidant mito-tempo. Treating HepG2 with mito-tempo significantly improved cell viability for PFCAs after 3 h. However, after 24 h, a significant increase in cell viability is only observed for PFOA. For PFSAs, mito-tempo did not significantly improve cell viability after 3 h and only improved cell viability for after 24 h.

**Fig. 10:**
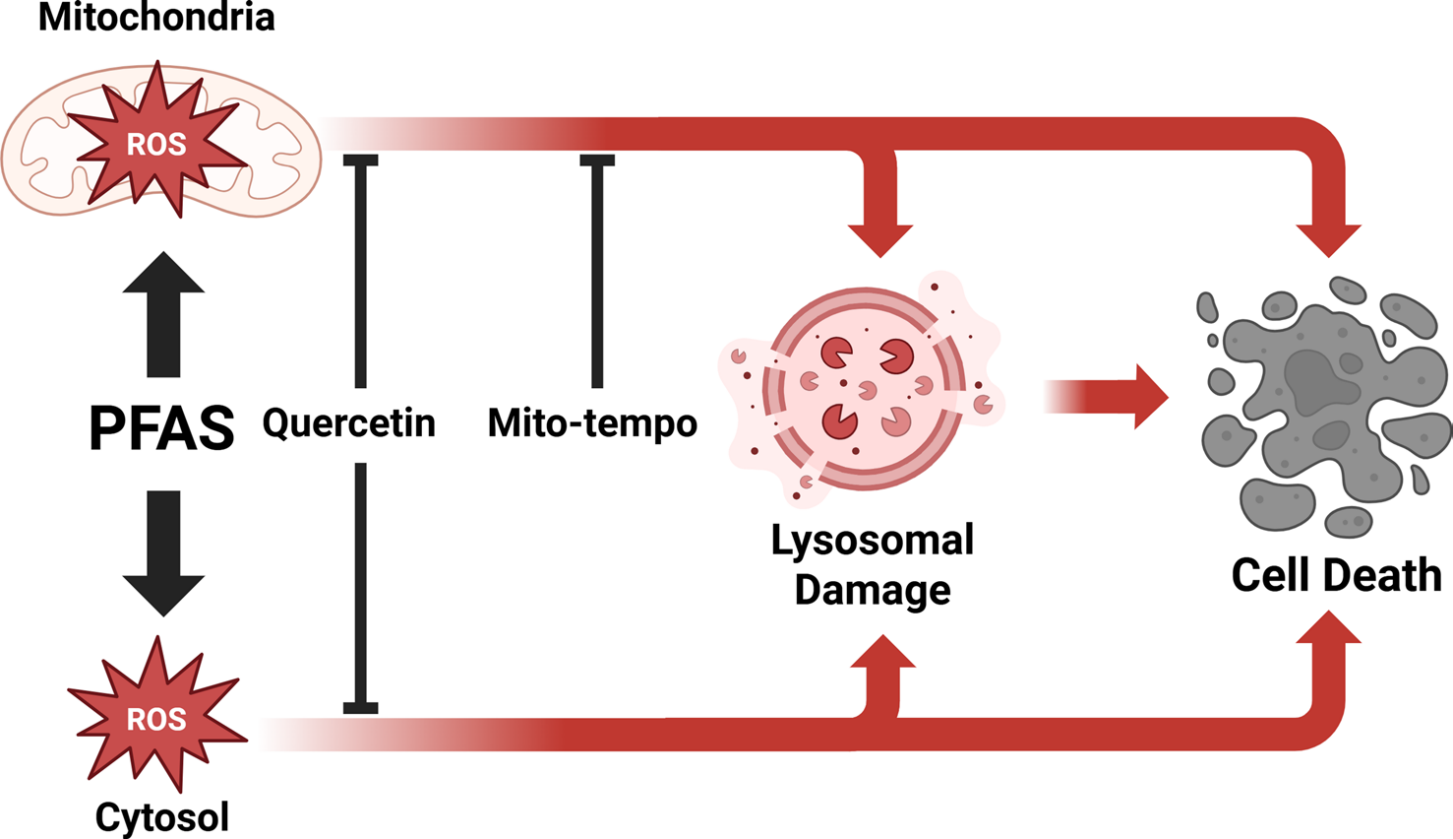
Effect of PFASs and antioxidants treatment on ROS generation and cell viability. Mito-tempo specifically targets mitochondrial ROS, while quercetin can rescue both mitochondrial and cytosolic ROS. Both antioxidants have been effective, indicating that ROS generation after exposure to PFASs comes from both compartments. Moreover, remediating ROS generation with antioxidants increased cell viability, demonstrating the importance of ROS-mediated toxicity for PFASs. However, lysosomal leakage can be reduced after exposure to either quercetin or mito-tempo. As such, the increase in lysosomal leakage results from ROS generation and is not due to PFASs directly interacting with the lysosome’s membrane, increasing its permeability.

Our data demonstrated the effectiveness of the antioxidants in remediating ROS generation and indicated that the mitochondria might be an important source of ROS for both PFCAs and PFSAs. However, while antioxidants improved cell viability, corroborating previous studies that demonstrated the role of ROS generation in cellular apoptosis induced by PFASs, it did not completely remediate the effect of PFASs exposure^20,21,31^. Notably, while quercetin has been effective in improving cell viability for PFCAs and PFSAs, mito-tempo had little effect on improving cell viability for PFCAs and no effect on improving cell viability for PFSAs. While the present results do not contradict previous publications on PFOA-induced apoptosis through ROS formation^28^, they outline a potential difference in the mechanism of action between PFOS and PFOA toxicity. Mito-tempo has been notably effective at reducing ROS generation yet did not improve cell viability as effectively as quercetin (Fig. 10). Therefore, while mitochondrial ROS generation increases due to PFSAs exposure, it was not strongly linked to cell viability. Another source of ROS might be key to PFSAs toxicity, through a different mechanism from that of PFCAs.

### Lysosomal integrity

Of the potential sources of ROS, the first sub-cellular site assessed was the integrity of the lysosomal membrane. While usually not a main source of ROS, lysosomes have been known to potentially generate ROS either through the release of reactive iron or from disruption of their redox homeostasis^32^. This is specifically relevant as PFOA has potentially been found to disrupt the integrity of the lysosomal membrane^27^. However, lysosomal dysfunction might also be a result of oxidative stress^33^. Quercetin and mito-tempo have been employed to determine whether lysosomal membrane permeabilization is a source of cytosolic ROS or whether it is caused by previously generated ROS.

While the present data confirmed that exposure to PFCAs and PFSAs can damage the membrane of the lysosomes (Fig. 5), treatment with either quercetin or mito-tempo significantly decreased lysosomal leaking for all PFASs. There were also no significant differences between PFOS and PFOA, indicating that the observed effect was independent of these PFASs headgroups. FTOHs did not affect lysosomal leakage, indicating that lysosomal leaking only occurred for PFASs generating ROS in HepG2. Moreover, lysosomal leakage could be rescued by pre-treatment with an antioxidant. Therefore, the damage to the lysosome membranes appears to result from increased ROS levels, not a causal factor (Fig. 10).

### Lipid peroxidation

While lipid peroxidation typically follows exposure to ROS or reactive iron species, it might also occur due to the denaturation of lipids present in cell membranes by chemical compounds, leading to further production of ROS^34,35^. As such, the effect of PFASs on lipid peroxidation was assessed as a potential alternative to the more classical pathway of ROS generation (Fig. 7). While no significant increase in lipid peroxidation was observed for PFOA, PFOS, and 6:2 FTOH after 3 h of exposure, a significant concentration-dependent increase was observed for PFOA and PFOS after 24 h. This indicates that the lipid peroxidation resulted from previously generated ROS and not from a more direct reaction between PFASs and lipid membranes that might subsequently generate ROS. PFOA led to a higher lipid peroxidation than PFOS. Those results were surprising as PFOS seemed to be a marginally more potent ROS generator than PFOA and, therefore, was expected to cause either a similar or larger amount of TBARS^22^.

### Mitochondrial activity

As exposure to PFASs has been linked to mitochondrial dysfunction, their effect on mitochondrial respiration has been assessed (Fig. 8)^21,28,31^. No proton leak was observed, so ROS generation does not appear to be caused by the permeabilization of the mitochondrial membrane, releasing reactive hydrogen protons to the cytosol. Similarly, no increase in ATP production was observed, which could be linked to ROS generation.

An unexpected increase in spare capacity was observed after PFCAs and PFSAs, as ROS generation is generally associated with decreased spare capacity^36^. However, the discrepancy can be explained by PFASs ability to increase MAPK-related signaling pathway, which in turn can greatly increase mitochondrial spare capacity^37,38^. Alternatively, increased mitochondrial spare capacity indicates a potential increase in oxidative phosphorylation, thus leading to increased intracellular ROS if not properly compensated by endogenous antioxidants^27^. Finally, a moderate increase in mitochondrial respiration has been measured for all tested PFCAs and PFSAs. Overall, the seahorse assay results indicate that PFASs exposure marginally increased mitochondrial activity, which might lead to increased ROS formation due to their redox activity, but not from proton leaks in the cytosol.

However, the data did not demonstrate a clear headgroup effect, with either a slightly stronger effect on the mitochondria from PFCAs, or no difference between PFCAs and PFSAs. Those results were unexpected as a previous study using HepG2 cells indicated that PFSAs have a slightly greater ROS generation potential than PFCAs^22^. At the same time, disruption of the mitochondrial energy homeostasis following PFASs exposure might be a compounding factor to ROS generation through increased redox activity, and it is unlikely to be the causal factor. Moreover, the present findings are consistent with research suggesting that mitochondrial dysfunction in HepG2 cells following exposure to PFASs is a result of oxidative stress rather than the source itself^39^.

### Peroxisome function

Finally, the effect of PFASs on peroxisome function was assessed as peroxisomes are one of the most important sources of ROS in the cell, along with the mitochondria^40^. As part of normal cell metabolism, hydrogen peroxide is generated as a by-product of β-oxidation, similar to the mitochondria, which in turn is broken down into water and oxygen by catalase. As such, when the normal functioning of peroxisomes is disrupted, it may lead to ROS generation^41^. First, the PFOA, PFOS, and 6:2 FTOH exposure effects were tested on β-oxidation (Figure S2). While PFASs exposure increased mitochondrial spare capacity in HepG2, thereby increasing redox reactions, they did not increase ROS generation through peroxisome activity.

Interestingly, both PFOA and PFOS decreased catalase activity (Fig. 9), while 6:2 FTOH did not cause any significant change. The present results indicate that PFASs can decrease the effectiveness of catalase, thereby decreasing the amount of ROS normally broken down by it and, thus, increasing ROS levels. Previous studies have shown that PFOA and PFOS may decrease the effectiveness of endogenous antioxidants. In mouse primary hepatocytes, PFOS and PFOA reduced superoxide dismutase 1 effectiveness^42^. In HepG2 cells, PFOA, PFNA, PFDA, and PFOS have been shown to deplete GHS levels^43^. As such, the reduced catalase activity is congruent with previous studies. However, while increased ACOX1 activity was observed in rodents, this was not the case for HepG2, which may be due to differences in gene regulation by PPARα between humans and rodents^39,44^. It appears that ROS generation induced by PFASs exposure may result from the depletion of endogenous antioxidants.

## Conclusion

The present study demonstrated that ROS generation caused by PFASs exposure is linked to reduced cell viability after acute exposure and showed the cellular sources of ROS in HepG2 cells. PFASs did not directly impact peroxisome activity but increased mitochondrial oxygen consumption. Moreover, the disruption of the lysosome membrane and lipid peroxidation after PFASs exposure resulted from ROS generation. PFOA and PFOS reduced catalase activity, demonstrating endogenous antioxidant depletion as a potential explanation for PFAS-related ROS generation. The present study outlines potential differences in the mechanism leading to cell death between PFCAs and PFSAs. Mito-tempo was as effective as quercetin in preventing cell death after PFCAs exposure, demonstrating that ROS from the mitochondria is key to PFCAs toxicity. However, for PFSAs, mito-tempo was significantly less effective than quercetin, indicating a cytosolic origin of the ROS. While PFOA and PFOS share some chemical characteristics, the sulfonic headgroup is potentially less polar than the carboxylic headgroup at physiological pH^45^. This corroborates with a previous study, which demonstrated that PFOA mitotoxicity was tied to the polarity of its headgroup^46^. Therefore, it appears that the polarity of the PFASs significantly affects its toxicity. The effect of the carbon chain-length appears consistent for both headgroups, with longer chain-lengths having stronger effects. Those results demonstrate a difference in the mode of action for PFOS compared to other PFASs, raising potential difficulties for the development of quantitative structure–activity models if there are compound-specific mechanisms of toxicity and warrant further studies on the effect of headgroups on the toxicity of PFASs.

## Supplementary Information

Supplementary Information 1.

Supplementary Information 2.

## Data Availability

The datasets generated and analyzed during the current study are available from the corresponding author upon reasonable request.
