# Supplementary material for: A subcellular study on reactive oxygen species generation by PFAS in HepG2 cells
Source: Sci Rep. 2025 Jul 1;15:21914. doi: 10.1038/s41598-025-07503-7 (PMC12215884; doi:10.1038/s41598-025-07503-7)
Supplement: Supplementary file 1 — Supplementary Information 1. [file 41598_2025_7503_MOESM1_ESM.pdf]

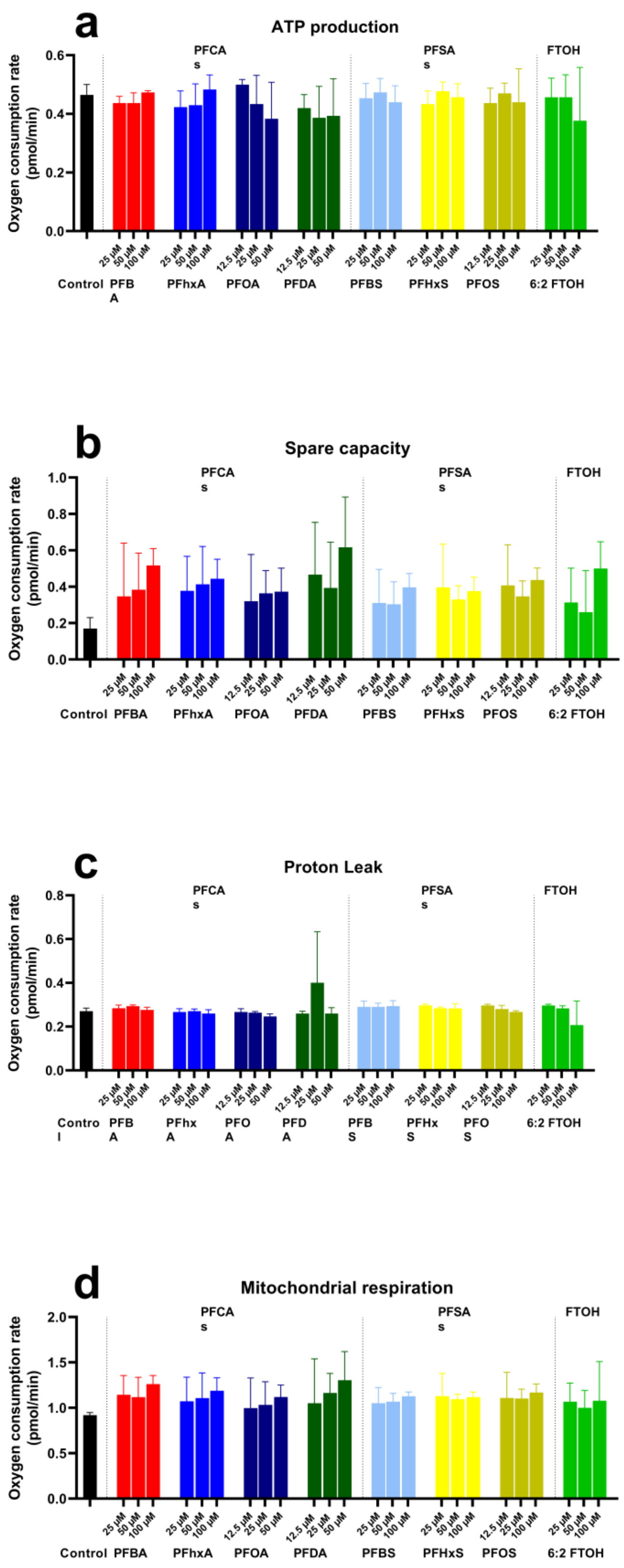

Figure S1: The effect of the different PFASs on HepG2 mitochondrial functioning after 3 hours of exposure. Mitochondrial ATP production (a), spare respiratory capacity (b), proton leak (c), and overall mitochondrial respiration (d) are shown. The Y-axis represents the oxygen consumption rate (pmol/min). Each condition was tested in triplicate. Data are expressed as mean ± SD.

V.H. Amstutz
Department of Pharmacology & Toxicology, Faculty of Medicine, Health & Life Science

PO Box 616, Maastricht, 6200 MD, The Netherlands

Email: v.amstutz@maastrichtuniversity.nl

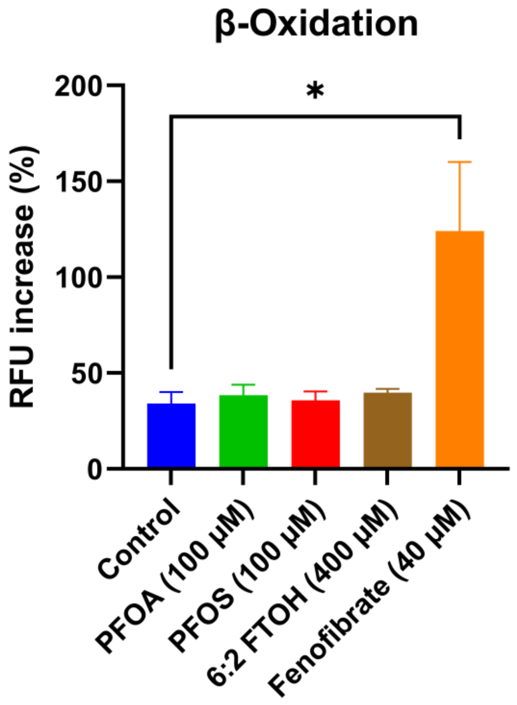

Figure S2: The effect of different PFASs on peroxisomal β-oxidation after 24 hours exposure. The control condition corresponds to cell culture medium with 0.2% DMSO. The Y-axis corresponds to the relative fluorescence increase ratio to samples without substrate. Each condition was tested in triplicate. Data are expressed as mean ± SD. * = $p < 0.05$.

V.H. Amstutz
Department of Pharmacology & Toxicology, Faculty of Medicine, Health & Life Science

PO Box 616, Maastricht, 6200 MD, The Netherlands

Email: v.amstutz@maastrichtuniversity.nl
